# The mediating effect of stigma between self-perceived burden and loneliness in stroke patients

**DOI:** 10.3389/fpsyt.2023.1219805

**Published:** 2023-07-27

**Authors:** Wenfeng Fan, Ke ke Ma, Cai xia Yang, Yuan li Guo

**Affiliations:** ^1^Nursing and Health School, Zhengzhou University, Zhengzhou, China; ^2^Neurology of the First Affiliated Hospital of Zhengzhou University, Zhengzhou, China

**Keywords:** stroke, self-perceived burden, stigma, loneliness, mediation

## Abstract

**Introduction:**

Stroke patients may experience reduced socialization and feelings of isolation due to post-stroke sequelae such as impaired motor function and cognitive deficits. Factors associated with loneliness need to be explored to develop targeted interventions. However, little is known about the impact of self-perceived burden and illness stigma on loneliness in this population.The aim of this study was to explore the mediating effect of stigma on self-perceived burden and loneliness in stroke patients.

**Methods:**

The cluster random sampling method was adopted to select 1028 stroke patients from the neurology department of third-grade A hospitals and second-grade A hospitals in 5 cities of Henan Province from May 2022 to August 2022. A general data questionnaire, self-perceived burden scale, stroke stigma scale, and loneliness scale were used to investigate. The structural equation model was used to analyze the mediating effect of stigma between self-perceived burden and stigma.

**Results:**

The loneliness of stroke patients was positively correlated with self-perceived burden and stigma. The results of the mediation analysis showed that stigma played a complete mediating role between self-perceived burden and loneliness.

**Discussion:**

The results of the study revealed the relationship between self-perceived burden, stigma, and loneliness in stroke patients. Stigma mediated the relationship between self-perceived burden and loneliness in this population.Stigma should be emphasized as an important modifiable psychological factor that affects loneliness of stroke patients.

## Introduction

Stroke is an acute cerebrovascular disease characterized by symptoms and signs of ischemic or hemorrhagic damage to brain tissue (1). Stroke has a prevalence of 1,115 cases per 100,000 people in China and a mortality rate of 115 cases per 100,000 people, with more than 2 million new cases each year. Nearly 50% of stroke patients exhibit motor dysfunction after a stroke (2), while cognitive deficits are present in 30 to 40% of patients (3). These include hemiparesis, aphasia, dementia, etc., which severely affect the quality of life and social participation of patients. Long-term post-stroke rehabilitation places a significant burden on families and society in terms of medical costs. Furthermore, patients are dependent on family members for care due to their reduced ability to care for themselves (4, 5). In addition, patients are prone to depression, stigma, loneliness, and other psychological disorders. As one of the most serious psychological problems, loneliness after stroke has attracted growing attention in recent years. Loneliness not only increases the risk of stroke by 32%, but is also associated with an elevated risk of stroke recurrence (6, 7). On the other hand, a national study has shown that loneliness is present in 44% of stroke patients (8). Moreover, loneliness reduces the quality of life of stroke patients (9). Therefore, it is necessary to explore the factors affecting post-stroke loneliness in the Chinese sociocultural context in order to develop targeted interventions.

Loneliness is defined as the subjective, unpleasant experience of lack or loss of companionship (10). The effects of loneliness on individuals are multifaceted. First, loneliness can increase adrenaline secretion, strengthen vasoconstriction, increase vascular resistance, and can lead to hypertension (11), which is a risk factor for stroke (6). Hence, loneliness increases the risk of stroke. Loneliness also increases the risk of depression, anxiety, and cognitive decline (12–14), and has a negative impact on one’s physical and mental health. Finally, loneliness can lead to social withdrawal, aggressive behavior, and suicidal thoughts (11, 14), and severely impacts social interpersonal relationships. Therefore, identifying the factors that influence loneliness is essential to improve the emotional health needs of stroke patients.

Exploring the factors associated with loneliness can help develop targeted interventions. Many factors are associated with loneliness, including demographic, illness, and psychological factors. Previous research has revealed higher loneliness in divorced patients, female gender, older, with smaller families, and economically disadvantaged patients (8). These factors are primarily comprise demographics and disease-related factors, but cannot be easily modified by interventions. Previous studies have shown that self-perceived burden is an important factor influencing loneliness (15). Moreover, stigma is positively associated with loneliness (16). Although many studies have been conducted to explore the link between loneliness and self-perceived burden and stigma, the mechanisms underlying these three variables are unclear and require further research.

Self-perceived burden is defined as an empathic concern due to the impact of one’s illness and care needs on others, resulting in guilt, distress, responsibility, and a diminished sense of self (17). Self-perceived burden is a common psychological condition among individuals with chronic illnesses. Over the past years, the overall burden of stroke (including health, economic, and social costs) on individuals, families, and national healthcare systems has increased (18, 19). Compared to Western countries, China’s health care system is still inadequate and formal community care services remain underdeveloped (20). Most stroke patients return home after stabilization for financial reasons, and failure to receive professional stroke rehabilitation after discharge may lead to poor physical function. Stroke survivors receiving care from others, especially those with functional impairment, demonstrate self-perceived burden (21). Influenced by Confucianism and the core values of collectivism, Chinese people regard caring for family members as an obligation. One of the core elements of Confucianism is filial piety, which includes the values of respect, love, and support for parents (20). Influenced by this traditional culture, family members of stroke patients in China take on the primary care of the patient. The collectivist sense of value emphasizes group interests over individual interests and suggests individual sacrifice for the sake of the family (20, 22, 23). As a result, stroke patients experience psychological stress when receiving care from family members because they do not want to become a burden or a liability to the family. Thus, Chinese familial values are both a source of social support and a possible source of stress for patients. Previous studies have reported that 99% of stroke patients experienced mild to moderate self-perceived burden (24), which was associated with physical conditions (e.g., pain and somatic weakness) and negative psychological conditions (e.g., depression and suicidal ideation) (25). In a study of older stroke patients, self-perceived burden was found to be negatively associated with patients’ self-management behaviors, negatively impacting their recovery and quality of life (26). In addition, a study reported a positive association between self-perceived burden and stigma in adults with epilepsy (27). Goffman was the first to suggest that stigma, which can “largely tarnish a person’s reputation,” induces psychological and emotional stress (5). Stigma is “an attribute that is deeply discrediting” and mainly involves psychological and emotional stress. Stigma hinders treatment and negatively impacts the quality of life of patients with chronic diseases (5). Influenced by Chinese family values, patients have a strong sense of dependence on their families (20), and studies have shown that high dependence leads to elevated levels of stigma (5). Stroke patients face varying degrees of physical, psychosocial, and cognitive impairment, such as hemiplegia, aphasia, and depression, which can further lead to feelings of shame (28), and can lead to a sense of stigma. Stigma and loneliness are positively correlated, which has been observed across countries and diseases. Self-perceived burden is an important factor influencing loneliness, and stigma may mediate this psychological process. The psychological stress theory (29) states that individuals who are exposed to negative life events (stressors) experience physiological, psychological, and behavioral stress responses mediated by a variety of internal and external factors. In this study, the self-perceived burden, stigma, and loneliness of stroke patients were analyzed in relation to the psychological stress theory. Stroke patients were hypothesized to have varying degrees of self-perceived burden after the onset of stroke, which might be mediated by the patient’s own psychological adjustment to developing stigma and result in increased loneliness. Therefore, structural equations were used to test this hypothesis, evaluating the mediating role of stigma in the relationship between self-perceived burden and loneliness.

In summary, self-perceived burden was hypothesized to be related to stigma and loneliness, and that stigma may be a potential mediator between self-perceived burden and loneliness. This study aimed to quantify the levels of self-perceived burden, stigma, and loneliness in stroke patients and to explore the potential relationship between self-perceived burden and stigma, and loneliness. The results would provide a theoretical basis for developing interventions to improve loneliness in such patients. This study hypothesized that (a) self-perceived burden is positively associated with loneliness (b), stigma is positively associated with loneliness, and (c) stigma mediates the relationship between self-perceived burden and loneliness.

## Methods

### Design

A descriptive cross-sectional design was utilized in this study, which followed the guidelines of the Strengthening Reports of Observational Studies in Epidemiology (STROBE) for cross-sectional studies.

### Participants

Using a convenience sampling method, the participants were recruited from 10 communities and 20 hospitals in 5 cities across eastern (Zhoukou City), western (Luoyang City), southern (Xinyang City) northern (Anyang City), and central (Zhengzhou City) Henan Province, China. Inclusion criteria: (1) age ≥ 18 years; (2) meeting the stroke diagnostic criteria; (3) being conscious and able to communicate effectively verbally or in writing; and (4) informed consent and voluntary participation in this study. Exclusion criteria: (1) those with a history of psychiatric disorders; (2) comorbidities including serious physical diseases, malignant tumors, or serious central nervous system diseases; and (3) autism diagnosed before the onset of stroke.

The sample size was taken to be 5–10 times the number of variables (30). A total of 25 variables were included in this study and a minimum sample size of 125 was calculated. Considering a 20% no-response rate, the minimum sample size for this study was 157.

### Data collection

Data were collected between May and August 2022 using self-report questionnaires, including demographic and clinical information, self-perceived burden, stigma, and loneliness. Stroke patients from the hospital completed the questionnaire on the day before discharge, and participants from the community provided data during the high-risk stroke population screening process. Surveyors were trained to use uniform instructional language to introduce the requirements for completing the questionnaire, informing participants of the purpose of the study and the right not to participate or to withdraw from the survey at any time. Paper or online questionnaires were distributed on-site, and the participants were asked to complete the questionnaires according to their actual situations. The average time to complete the questionnaire was approximately 25 min. The questionnaires were verified and collected on the spot after completion. A total of 1,040 questionnaires were distributed, with 1,028 valid questionnaires, achieving a valid recovery rate of 97.2%.

### Tools

#### Demographic and clinical information

The demographic and clinical information included gender, age, education level, place of residence, economic status, marital status, number of children, etc.

#### Self-perceived burden scale

The Self-Perceived Burden Scale (17) (SPBS) was used to assess patients’ self-perceived burden. The scale consisted of 10 items, evaluating 3 dimensions: physical burden, emotional burden, and financial burden. A 5-point Likert scale was used, with a total score range of 10–50. Higher scores indicated a higher self-perceived burden. The total score was classified into four levels: none (10–19), mild (20–29), moderate (30–39), and severe (40–50). Previous studies have indicated a Cronbach’s alpha coefficient of 0.86 for this scale, while a Cronbach’s alpha coefficient of 0.926 was achieved in the current study.

#### Stroke stigma scale

Stigma was assessed by the Stroke Stigma Scale (SSS) (31). The scale includes self-perception (5 entries), somatic impairment (4 entries), experience of discrimination (4 entries), and social interaction (3 entries), for a total of 16 entries across 4 dimensions. All were scored on a 5-point Likert scale (1 = never, 5 = always). The total score ranged from 16 to 80, with higher scores indicating higher levels of stigma among the subjects. The total score was divided into five levels: very low (<18.75), low (18.75–31.25), moderate (31.25–48.44), high (48.44–68.75), and very high (>68.75). The Cronbach’s alpha coefficient for each dimension ranged between 0.771–0.864.The Cronbach’s alpha coefficient of this scale in this study was 0.852.

#### Loneliness scale

The UCLA (University of California, Los Angeles) Loneliness Scale (32) was used to assess loneliness, which consisted of 20 items. Each of the items was rated on a 4-point Likert scale (1–4 representing never to always, respectively). The total score was 20–80, with higher scores indicating more severe loneliness. The scale was divided into four levels: low (20–34), moderate (35–49), moderate to high (50–64), and high (65–80). Previous studies have indicated a Cronbach’s alpha coefficient of 0.89 for this scale, while a Cronbach’s alpha coefficient of 0.825 was achieved in the current study.

### Ethical considerations

The study protocol was approved by the Ethics Committee of the First Affiliated Hospital of Zhengzhou University (2020-KY-459). The study followed the principles of anonymity and confidentiality, and informed consent was obtained from the participants.

### Data analysis

The SPSSAU online data analysis platform^1^ was used for data analysis. Count data were described by frequency and composition ratio, whereas measurement data were described by x̄ ± SD and compared between groups using independent samples *t*-test and one-way ANOVA. The measurement data that did not conform to a normal distribution were described by median and quartiles, and the Mann–Whitney *U* test and Kruskal–Wallis *H* test were used for comparison between groups. Pearson correlation analysis was used to explore the correlation between self-perceived burden, stigma, and loneliness in stroke patients. The pathways of action among self-perceived burden, stigma, and loneliness were analyzed using SPSSAU, and indirect effects were estimated by the bootstrap resampling method with 5,000 replicate samples, and 95% confidence intervals (CI) were calculated. A mediating effect was considered significant if the 95% CI did not include zero. *p* < 0.05 (two-sided test) was considered statistically significant.

## Results

### Participant characteristics

A total of 1,028 stroke patients were included in this study, and the mean age of the patients was (62.09 ± 13.72) years. There were 578 (56.2%) males and 450 (43.8%) females, 85.6% of the patients were married (880), 656 (63.8%) patients lived in rural areas, 73.3% of the participants had a low level of education (secondary school or less), and 83.9% had more than 2 children. The baseline characteristics are displayed in Table 1.

**Table 1:** Participant characteristics (*N* = 1,028).

Characteristic	*N* (%)	UCLA score [score, M(P25,P75)]	*Z*/*H*	*p*
Age (years)			3.915^(1)^	0.271
≤ 54	271(26.4)	23(18,27)		
55 ~ 64	261(25.4)	22(18,27)		
65 ~ 71	239(23.2)	23(19,29)		
≥ 72	257(25)	23(19,28)		
Sex			−0.267^(2)^	0.789
Male	578(56.2)	23(18,28)		
Female	450(43.8)	23(18,28)		
Educational level			13.637^(1)^	0.003
≤ Primary school	421(41.0)	24(20,28)		
Middle school	332(32.3)	23(17,27)		
High school	142(13.8)	22(17.5,27)		
College or university	133(12.9)	21(15.5,26)		
Employment			4.338^(1)^	0.114
Unemployed	626(60.9)	23(19,28)		
Retire	229(22.3)	23(17,29)		
On the job	173(16.8)	23(17,26.75)		
Resident			−1.653^(2)^	0.098
Countryside	656(63.8)	23(19,28)		
City	372(36.2)	22(17,28)		
Live alone			−0.209^(2)^	0.835
Yes	120(11.7)	23(18,28)		
No	908(88.3)	23(18.75,28)		
Marry status			1.821^(1)^	0.610
Unmarried	42(4.1)	23(17,30)		
Married	880(85.6)	23(18,28)		
Divorce	14(1.4)	25(20.75,29.25)		
Widowhood	92(8.9)	22(18.25,27.75)		
Income (CNY/monthly)			10.612^(1)^	0.014
< 3,000	530(51.6)	23(19,29)		
3,000 ≤ income < 5,000	425(41.3)	22(18,27)		
5,000 ≤ income < 10,000	62(6.0)	23(14.75,26)		
≥ 10,000	11(1.1)	21(11,25)		
Smoke			−2.502^(2)^	0.012
Yes	296(28.8)	22(18,26)		
No	732(71.2)	23(19,28)		
Drink			−1.797^(2)^	0.072
Yes	251(24.4)	22(18,27)		
No	777(75.6)	23(19,28)		
Child-number			4.653^(1)^	0.098
0	39(3.8)	26(20,31)		
1 ~ 3	838(81.5)	23(18,28)		
≥ 4	151(14.7)	23(20,27)		
Stroke type			−2.436^(2)^	0.015
Ischemic	843(82.0)	23(18,27)		
Hemorrhagic	185(18)	23.5(20,30)		
Family history of stroke			−0.873^(2)^	0.383
Yes	156(15.2)	23(20,27)		
No	872(84.8)	23(18,28)		
Recur-times			6.700^(1)^	0.082
0	296(28.8)	22(17,28)		
1	508(49.4)	23(19,27)		
2	166(16.2)	24(18,29)		
≥ 3	58(5.6)	24(18.75,28)		
Disease duration (months)			12.515^(1)^	0.014
0 < months≤3	749(72.9)	23(18,28)		
3 < months≤6	114(11.1)	25(20.75,29)		
6 < months ≤ 9	29(2.8)	24(21,29.5)		
9 < months ≤ 12	26(2.5)	19.5(16.75,26.25)		
>12	110(10.7)	22.5(18,26)		
Thrombolysis			−0.787^(2)^	0.431
Yes	136(13.2)	22(16,28)		
No	892(86.8)	23(19,28)		
Hypertension			−1.050^(2)^	0.294
Yes	323(31.4)	22(18,28)		
No	705(68.6)	23(19,28)		
Diabetes			−1.224^(2)^	0.221
Yes	759(73.8)	23(19,28)		
No	269(26.2)	23(18,27)		
Hyperlipidemia			−1.710^(2)^	0.087
Yes	837(81.4)	23(18,27)		
No	191(18.6)	24(18,29)		
Heart disease			−1.997^(2)^	0.046
Yes	854(83.1)	23(18,27)		
No	174(16.9)	24(19,29)		
NHISS score			1.519^(1)^	0.218
<5	628(61.1)	22(18,27)		
5 ~ 15	303(29.5)	23(19,28)		
16 ~ 20	62(6.0)	26(21,29)		
>20	35(3.4)	27(21,31)		
mRS grade			9.121^(1)^	0.104
0	228(22.2)	23(19,28)		
1	275(26.8)	22(17,28)		
2	188(18.3)	23(19.25,29)		
3	178(17.3)	23(18,28)		
4	102(9.9)	23.5(21,28.25)		
5	57(5.5)	22(15,28)		
Total	1,028			

(1) *H*-value; (2) *Z*-value.

### Correlation analysis between variables

The results of this study showed that all three variables, self-perceived burden, stigma, and loneliness, were positively correlated with each other. The mean loneliness score in stroke patients in this study was (22.86 ± 6.57), while the mean score of self-perceived burden was (20.46 ± 6.16), and the mean score of stigma was (29.16 ± 8.13), as displayed in Table 2. 70% of the responses indicated low levels of loneliness, and 1.3% suggested moderate levels of loneliness. In contrast, high and higher levels of loneliness were not detected. The percentages of insignificant, mild, and moderate self-perceived burden were 40.3, 53.6, and 6.1%, respectively. Furthermore, the percentages of patients with very low, low, moderate, and high levels of stigma were 8.2, 53.5, 36.7, and 1.7%, respectively. The correlation analysis is shown in Table 2, where loneliness was positively correlated with both self-perceived burden (*r* = 0.254, *p* < 0.01) and stigma (*r* = 0.604, *p* < 0.01).

**Table 2:** Correlation analysis of self-perceived burden, stigma, and loneliness.

	^–^X ± SD	Loneliness	Stigma	Self-perceived burden
Loneliness	22.86 ± 6.568	1		
Stigma	29.16 ± 8.125	0.582^**^	1	
Self-perceived burden	20.46 ± 6.161	0.275^**^	0.472^**^	1

** *p* < 0.01.

### Intermediary analysis

**Figure 1:**
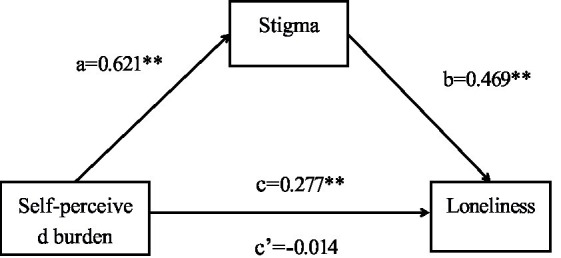
Mediating effect of stigma between self-perceived burden and loneliness a, effect of self-perceived burden on stigma; b, effect of stigma on loneliness; c, total effect of self-perceived burden on loneliness; c’, direct effect of self-perceived burden on loneliness; ^**^p < 0.01.

The results of this study show that stigma fully mediates the relationship between self-perceived burden and loneliness. In other words, self-perceived burden may exacerbate patients’ stigma and result in increased loneliness. A structural equation model was constructed using the SPSSAU data analysis platform; variables with differences (*p* < 0.05) in scores on the loneliness scale in education, family monthly income, smoking status, type of stroke, duration of illness, and heart disease were used as control variables. Self-perceived burden was set as the independent variable, with stigma as the mediating variable and loneliness as the dependent variable, and path analysis was used to fit the hypothetical model. The model fitting results revealed that the chi-squared degrees of freedom ratio showed a good model fit, with the chi-square freedom ratio (*χ*^2^/*df*) = 0.000, the fitness index (GFI) = 1.000, the benchmarked fitness index (NFI) = 1.000, the comparative fitness index (CFI) = 1.001, and the asymptotic residual mean square and root square (RMSEA) = 0.000, as detailed in Table 3 and Figure 1. The results showed that self-perceived burden significantly predicted stigma (*β* = 0.621, *t* = 16.898, *p <* 0.001), and stigma significantly predicted loneliness (*β* = 0.469, *t* = 20.331, *p* < 0.001). In contrast, the direct effect of self-perceived burden on loneliness was not significant (*β* = −0.014, *t* = −0.452, *p* > 0.05), ab = 0.291, Boot SE = 0.023, 95% confidence interval (0.229–0.320), indicating that stigma fully mediates the effect between self-perceived burden and loneliness, as displayed in Table 4.

**Table 3:** Fitting indicators and evaluation criteria.

Fit index	*χ*^2^/*df*	GFI	RMSEA	NFI	IFI	CFI
Evaluation criteria	<3	>0.90	<0.10	<0.90	>0.90	>0.90
Index value	0.000	1.000	0.000	1.000	1.001	1.001

*χ*^2^/*df*, Chi-square/degree of freedom; GFI, goodness-of-fit index; RMSEA, root mean square error of approximation; NFI, normed fit index; IFI, incremental fit index; CFI, comparative fit index.

**Table 4:** Mediating effect of stigma between self-perceived burden and loneliness.

	Loneliness			Stigma			Loneliness		
	*β*	SE	*t*	*β*	SE	*t*	*β*	SE	*t*
intercept	15.288^**^	1.359	11.246	15.732^**^	1.557	10.103	7.910^**^	1.203	6.574
Education	−0.364	0.214	−1.702	0.152	0.245	0.62	−0.436^*^	0.181	−2.411
Income	−0.505	0.335	−1.508	−0.184	0.384	−0.479	−0.419	0.283	−1.482
Smoke	1.025^*^	0.434	2.363	−0.073	0.497	−0.147	1.059^**^	0.366	2.894
Stroke type	1.328^*^	0.516	2.572	0.534	0.592	0.904	1.078^*^	0.436	2.472
Disease duration	−0.024	0.151	−0.161	0.049	0.173	0.284	−0.047	0.127	−0.372
Heart disease	0.795	0.532	1.494	0.747	0.61	1.225	0.445	0.449	0.99
SPB	0.277^**^	0.032	8.646	0.621^**^	0.037	16.898	−0.014	0.031	−0.452
Stigma	–	–	–	–	–	–	0.469^**^	0.023	20.331
*R*^2^	0.096			0.225			0.357		
*F*	15.538^**^			42.323^**^			0.759^**^		

SPB, self-perceived burden; β, standardized regression coefficient; SE, standard error; ^*^p < 0.05, ^**^p < 0.01.

## Discussion

Understanding the psychological mediators of the relationship between self-perceived burdens and loneliness is crucial for developing strategies to improve loneliness in stroke patients. Previous studies have only explored the relationship between loneliness, self-perception, and stigma separately, but stigma has been neglected as a mediator. To our knowledge, this is the first study investigating the relationship between self-perceived burden, stigma, and loneliness in stroke patients, using a large hospital and community-based sample. This study confirmed our hypothesis, revealing a positive association between self-perceived burden and stigma and loneliness. More importantly, stigma fully mediated the relationship between self-perceived burden and loneliness. Our findings provide directions for the development of interventions to improve loneliness in such patients.

The mean loneliness scores indicated low levels of loneliness in stroke patients, which were even lower than stroke patients in the United States (33). This finding could be attributed to cultural differences. Individual independence is a dominant ideology in the United States, whereas collectivism plays a central role in China. The typical American family is a “mobile family,” as they enjoy moving from place to place very often. Chinese people with collectivist values tend to live in a fixed place and maintain close ties with their neighbors and help each other (34), which may influence loneliness. In this study, the self-perceived burden score of stroke patients was (20.46 ± 6.16), similar to the findings of Ren et al. (35), which were lower than epilepsy patients and cervical cancer patients undergoing radiotherapy (27, 36). Self-perceived burden was present in 59.7% of stroke patients in this study, and about 90.2% reported low to moderate stigma, emphasizing the high incidence of self-perceived burden and stigma. Many Chinese people believe in the concept of karma (37). The Taoist view of good and evil states that the world is essentially fair and that good is rewarded with good and evil with evil. Therefore, influenced by traditional culture, stroke patients may believe that the suffering caused by the disease is self-inflicted, which might increase the patient’s self-perceived burden and stigma. The risk of recurrent stroke is high (5-year incidence 32.3%) (38), which can be disabling or fatal. Disease recurrence can cause significant psychological stress and loss of confidence in treatment, leading to negative emotions and avoidant coping mechanisms, which may trigger feelings of self-perceived burden, stigma, and loneliness. Loneliness increases the 5-year mortality rate of stroke patients by three times (39) and is associated with lower self-esteem (6), which is detrimental to the physical and mental health of patients. Therefore, exploring the factors influencing loneliness in stroke patients provides a reference for strategies to improve patients’ loneliness and their prognosis.

Consistent with previous studies (36), the study found a positive association between self-perceived burden and loneliness. Stroke patients with higher self-perceived burden have higher levels of loneliness. In addition, people with high self-perceived burden are particularly sensitive to relying on others and exhibit decreased contact, leading to increased loneliness (15). In addition, most stroke patients have physical dysfunctions and require assistance with daily living activities, which may lead to feelings of guilt and burden, and thus increase their self-perceived burden (21, 35). In traditional Chinese culture, loss of self-care ability and dependence on others in daily life may cause patients to develop thoughts of uselessness, a reduced sense of self-worth, and worry about becoming a burden to the entire family (28). The interaction between self-perceived burden and physical activity limitations affects loneliness (40). Moreover, self-perceived burden is associated with negative psychological conditions, such as anxiety and depression (25). This may lead to higher levels of loneliness (11). Our findings suggest that self-perceived burden should be considered an important factor in improving loneliness in stroke patients.

This study suggests a positive correlation between stigma and loneliness in patients with stroke, that is, stroke patients with higher levels of stigma have higher levels of loneliness. Patients with chronic diseases often feel guilt and self-blame for current or previous unhealthy lifestyles, which, in addition to stigmatizing attitudes in social settings, may lead them to distance themselves from others and result in loneliness (41). Stroke patients with higher levels of stigma tend to choose to live in isolation at home (42), which may exacerbate the patient’s loneliness. Furthermore, physical dysfunctions are a common complication of stroke, including hemiplegia, aphasia, and salivation, and can lead to self-image disorders and stigma. Patients may be afraid of making a fool of themselves in front of others and reduce social interaction, resulting in loneliness. Chinese culture focuses mainly on interpersonal relationships. Chinese people are very sensitive to matters of reputation and will do their best to maintain it (22). Another characteristic of Chinese culture in terms of important social relationships is the close ties between neighbors. As the old Chinese proverb says, a close neighbor is better than a distant cousin, emphasizing the responsibility of neighbors to accompany and care for each other. However, this also means that news can spread very quickly, and stroke patients with a disturbed self-image may reduce interactions with those around them in order to maintain their reputation, leading to loneliness.

Expectedly, stigma mediates the relationship between self-perceived burden and loneliness in stroke patients. In other words, self-perceived burden may exacerbate patients’ stigma and, in turn, their loneliness. Additionally, a positive correlation between self-perceived burden and stigma has been shown (27). Self-perceived burden is a barrier to help-seeking (40). Therefore, stroke patients may adopt avoidant coping behaviors, aggravated by a low motivation to seek medical care, increased physical disability, and increased stigma (5). These aspects further increase their loneliness. However, the psychological factor of stigma can be modified by intervention, and our findings suggest that stigma is an actionable target for loneliness intervention programs for stroke patients.

### Limitations

Nevertheless, the limitations of the study should be acknowledged. First, owing to the cross-sectional design of this study and the application of mediation analysis, only correlations between the variables were demonstrated, while the causal relationships between self-perceived burden, stigma and loneliness could not be determined. A longitudinal study is needed to determine the causal relationship between the variables in the future. In addition, this study used a self-administered questionnaire to collect data, which may result in recall and reporting bias.

## Conclusion

In conclusion, this study shows that self-perceived burden and stigma are key correlates of loneliness in stroke patients, and that after controlling for the effects of confounders, stigma fully mediates the relationship between self-perceived burden and loneliness. This suggests that healthcare professionals should pay attention to the assessment of patient stigma in their clinical work. Appropriate measures should be taken to reduce patients’ stigma, such as educating stroke patients about the disease, relieving their psychological pressure, and encouraging patients to actively participate in social life and face the disease with a positive attitude to reduce negative emotions and loneliness.

### Relevance to clinical practice

The present study further confirms the high prevalence of loneliness in stroke patients. As loneliness affects the prognosis of stroke patients and significantly impacts their recovery, elucidating the factors that contribute to loneliness and exploring measures to reduce loneliness is essential in stroke patients. The results of this study suggest that self-perceived burden can indirectly influence loneliness in stroke patients due to the mediating role of stigma. This relationship entails some clinical implications. First, the assessment of stroke patients’ loneliness, self-perceived burden, and stigma should be emphasized. Patients’ negative emotions should be identified and promptly relieved to promote their mental health. Educational activities should be introduced to change people’s perceptions of the disease and reduce the public discrimination and the internalized stigma of patients. Second, the Chinese government should establish a robust social security system to provide comprehensive medical coverage and social welfare for stroke patients. For example, strengthening community stroke care systems and recruiting staff who match the cultural beliefs of the area in which they are located to provide culturally sensitive care. Finally, the development of the internet has facilitated communication and interaction, and tools such as social media can be used to connect stroke patients with others, promote social activity and participation, and assist their reintegration into society.

## Data availability statement

The raw data supporting the conclusions of this article will be made available by the authors, without undue reservation.

## Ethics statement

The studies involving human participants were reviewed and approved by the Ethics Committee of the First Affiliated Hospital of Zhengzhou University (2020-KY-459). Written informed consent to participate in this study was provided by the participants’ legal guardian/next of kin.

## Author contributions

YG, KM, and CY: designed the study. WF, KM, CY, and YG: gathered data. KM, YG, and WF: analyzed data. WF and KM: drafted the manuscript. KM, YG, and CY: revised the manuscript. All authors contributed to the article and approved the submitted version.

## Funding

The study was supported by Henan Science and Technology Research Project(222102310246).

## Conflict of interest

The authors declare that the research was conducted in the absence of any commercial or financial relationships that could be construed as a potential conflict of interest.

## Publisher’s note

All claims expressed in this article are solely those of the authors and do not necessarily represent those of their affiliated organizations, or those of the publisher, the editors and the reviewers. Any product that may be evaluated in this article, or claim that may be made by its manufacturer, is not guaranteed or endorsed by the publisher.

## Footnotes

^1^https://spssau.com/index.html
